# Altered Lnc-EGFR, SNHG1, and LincRNA-Cox2 Profiles in Patients with Relapsing-Remitting Multiple Sclerosis: Impact on Disease Activity and Progression

**DOI:** 10.3390/diagnostics13081448

**Published:** 2023-04-17

**Authors:** Mohamed S. Attia, Heba A. Ewida, Mohamed Aly Abdel Hafez, Shohda A. El-Maraghy, Maha M. El-Sawalhi

**Affiliations:** 1 Pharmacology, Toxicology and Biochemistry Department, Faculty of Pharmacy, Future University in Egypt (FUE), Cairo 11835, Egypt; mohamed.radwan@fue.edu.eg (M.S.A.); hebaallah.atef@fue.edu.eg (H.A.E.); 2 Neurology Department, Faculty of Medicine, Ain Shams University, Cairo 11566, Egypt; mohamedaly@med.asu.edu.eg; 3 Biochemistry Department, Faculty of Pharmacy, Cairo University, Cairo 11562, Egypt; shohda.elmaraghy@pharma.cu.edu.eg

**Keywords:** relapsing–remitting multiple sclerosis, lnc-EGFR, SNHG1, lincRNA-Cox2, FOXP3, NLRP3-inflammasome

## Abstract

Relapsing–remitting multiple sclerosis (RRMS) is the most prevalent MS subtype. Ample evidence has indicated that long noncoding RNAs (lncRNAs) are crucial players in autoimmune and inflammatory disorders. This study investigated the expression of lnc-EGFR, SNHG1, and lincRNA-Cox2 in RRMS patients during active relapses and in remission. Additionally, the expression of FOXP3, a master transcription factor for regulatory T cells, and NLRP3-inflammasome-related genes were determined. Relationships between these parameters and MS activity and annualized relapse rate (ARR) were also evaluated. The study included 100 Egyptian participants: 70 RRMS patients (35 during relapse and 35 in remission) and 30 healthy controls. RRMS patients showed significant downregulation of lnc-EGFR and FOXP3 and dramatic upregulation of SNHG1, lincRNA-Cox2, NLRP3, ASC, and caspase-1 compared to controls. Lower serum TGF-β1 and elevated IL-1β levels were observed in RRMS patients. Notably, patients during relapses displayed more significant alterations than those in remission. Lnc-EGFR was positively correlated with FOXP3 and TGF-β1 and negatively correlated with ARR, SNHG1, lincRNA-Cox2, and NLRP3 inflammasome components. Meanwhile, SNHG1 and lincRNA-Cox2 were positively correlated with ARR, NLRP3, ASC, caspase-1, and IL-1β. Excellent diagnostic performance for lnc-EGFR, FOXP3, and TGF-β1 was demonstrated, while all biomarkers exhibited strong prognostic potential for predicting relapses. Finally, the differential expression of lnc-EGFR, SNHG1, and lincRNA-Cox2 in RRMS patients, especially during relapses, suggests their involvement in RRMS pathogenesis and activity. Correlation between their expression and ARR implies relationships to disease progression. Our findings also highlight their promising roles as biomarkers for RRMS.

## 1. Introduction

Multiple sclerosis (MS) is a complex neurodegenerative disease with both autoimmune and inflammatory characteristics affecting the central nervous system (CNS). It is characterized by demyelination with incomplete remyelination and axonal injury, resulting in several sensory and motor impairments [1]. MS is the most common non-traumatic disabling disorder affecting young adults, with globally increasing incidence [2]. The most prevalent MS phenotype is relapsing–remitting MS (RRMS), affecting about 85% of MS patients. RRMS is characterized by relapses or episodes of transient exacerbations of neurological signs or symptoms followed by periods of remission or recovery with improvement or disappearance of symptoms. Some RRMS patients may gradually develop secondary progressive multiple sclerosis in which the disease course worsens without leveling off of symptom severity [3].

Despite intense research, the mechanisms governing a relapse incident still remain incompletely understood [4]. Therefore, a better understanding of RRMS development and prognosis as well as investigating novel biomarkers for disease activity might help in the improvement of disease management.

Traditionally, it has been believed that MS is caused by the activation of peripheral autoreactive, myelin-specific T cells that migrate into the CNS and cause inflammation, resulting in demyelination and the disease process initiation. Indeed, inflammatory T helper (Th) cells, such as interferon (IFN)-γ-producing Th1 cells and interleukin (IL)-17-producing Th17 cells have a well-known role in the pathogenesis of MS [1,5,6]. Conversely, CD4+ regulatory T (Treg) cells were identified as a negative regulator of T helper cell functions via secreting anti-inflammatory cytokines such as IL-4, IL-10, and tumor growth factor (TGF)-β, thus playing a pivotal role in the maintenance of self-tolerance and prevention of autoimmunity [1,5,6,7]. CD4+ Treg cells are characterized by the expression of several markers, including FOXP3, a forkhead/winged-helix transcription factor. Continuous expression of FOXP3 is known to be essential for proper development and maintenance of Treg cells’ suppressive functions [7,8]. Dysfunctions or impaired maturation of Treg cells have been demonstrated in animal models of MS [9]. Moreover, disrupted balance between inflammatory and regulatory T cells [10] and impaired suppressive functions of Treg cells have also been detected in patients with MS [11].

Neuroinflammation has been recognized as a crucial player in the pathogenesis and progression of various neurodegenerative diseases including MS. Accumulating evidence has demonstrated the association between inflammasome activation, especially NLRP3 (NOD-like receptor protein 3) and MS development [12]. The NLRP3 inflammasome is a multimeric protein complex that is a member of NOD-like receptors (NLRs) and consists of three different proteins: a protein that senses stimulation (NLRP3), an adapter protein (apoptosis-associated speck-like protein (ASC)), and a catalytic protein (procaspase-1) [13]. Inflammasome activation induces the assembly of these molecules into a complex that accumulates to form a large cytosolic oligomer, which allows the self-cleavage of pro-caspase-1 to the active form of caspase-1. Consequently, caspase-1 cleaves pro-IL-1β and pro-IL-18 to their physiologically active forms and induces a pro-inflammatory form of cell death, pyroptosis, with exacerbation of inflammation. Of note, inflammasome activation has been identified as a rapid, highly reactive, and potent amplifier of inflammation that is essential to immune function [14]. A number of studies using experimental autoimmune encephalomyelitis (EAE), an animal model of MS, demonstrated the direct involvement of NLRP3 inflammasome in the pathogenesis of MS [15,16,17]. Similarly, up-regulation of NLRP3, caspase-1, and IL-1β expression in peripheral blood mononuclear cells (PBMCs) [18] and increased caspase-1 and IL-1β proteins in PBMCs, serum, and cerebrospinal fluid (CSF) [19] have been found in MS patients. Moreover, serum caspase-1 and ASC levels were reported to be potential candidate biomarkers for MS onset [20].

Over the past decade, several reports have demonstrated the usefulness of serum RNAs as prognostic and diagnostic biomarkers in various human diseases including MS [21,22,23]. Furthermore, the importance of long noncoding RNAs (lncRNAs) in epigenetic regulation of a series of biological processes and their involvement in autoimmune [24] and inflammatory diseases [25] have been established. Although many MS-related lncRNAs have been sequenced and their functions have been characterized [26], and although dysregulated lncRNA expression has been found in serum, plasma, and PBMCs of patients with MS [21], the expression and the role of several other lncRNAs in MS still remains to be explored.

Lnc-epidermal growth factor receptor (lnc-EGFR) is a novel lncRNA that was identified by Jiang et al. [27]. The authors found that lnc-EGFR was highly expressed in Treg cells and positively correlated with FOXP3 expression in hepatocellular carcinoma (HCC). Notably, this study indicated that lnc-EGFR specifically binds to EGFR and prevents its ubiquitination, resulting in maintenance of EGFR activation, thus leading to the stimulation of Treg differentiation in HCC [27]. Interestingly, the involvement of lnc-EGFR in different neurodegenerative, autoimmune, and inflammatory disorders has not yet been revealed.

The long noncoding RNA small nucleolar RNA host gene 1 (SNHG1) is another newly identified lncRNA that was found to be upregulated in the brain specimens derived from patients with Parkinson’s disease (PD) [28] and in MPTP-induced PD mouse models [29]. Additionally, SNHG1 has been implicated in microglial activation and neuroinflammation [29,30]. Furthermore, SNHG1 overexpression was shown to be correlated with the activation of the NLRP3 inflammasome and enhanced expression of NLRP3, ASC, and cleaved caspase-1 in lipopolysaccharide-stimulated BV2 microglial cells in vitro [29]. A recent study using bioinformatics analysis indicated a higher level of SNHG1 in patients with RRMS in relation to the controls and suggested a possible role for SNHG1 in the pathogenesis of MS through an associated competing endogenous RNA axis [31]. Nevertheless, the authors recommended that these predicted findings need to be validated by confirmative experimental approaches.

Long intergenic RNA Cox2 (lincRNA-Cox2), the third lncRNA addressed herein, is an inflammatory inducible lncRNA that has acquired its name due to its location in relation to the cyclooxygenase-2 gene. LincRNA-Cox2 has been reported to be one of the best studied lncRNAs that can modulate the transcriptional control of inflammatory gene expression [25,32]. Additionally, lincRNA-Cox2 has been identified as a coactivator of NF-kB for the transcription of late-primary response genes involved in the innate immune response via epigenetic chromatin remodeling [24,33]. A recent elegant study showed that lincRNA-Cox2 can regulate NLRP3-inflammasome-mediated neuroinflammation and the expression of the inflammasome sensor NLRP3 and adaptor ASC. Furthermore, knockdown of lincRNA-Cox2 resulted in inhibition of inflammasome activation and prevention of lincRNA-Cox2-induced caspase-1 activation in macrophages and microglia cells in vitro as well as reduction in CNS inflammation and EAE severity in vivo [34]. Importantly, the role of lincRNA-Cox2 in RRMS and its possible contribution to disease activity have not yet been elucidated.

Based on these findings, our study aimed to investigate the expression profiles of the three lncRNAs (lnc-EGFR, SNHG1, and lincRNA-Cox2) in Egyptian patients with RRMS during active relapses and in remission in an attempt to assess their impact on MS activity and future progression. In addition, the expression of FOXP3, a master transcription factor in Treg cells and NLRP3-inflammasome-related genes, including NLRP3, ASC, and caspase-1, along with the serum levels of TGF-β1and IL-1β were determined. The possible correlations between the three lncRNAs, the clinical characteristics, and the biochemical parameters were also assessed. The clinical relevance of our biomarkers as novel diagnostic and prognostic tools for RRMS was evaluated.

## 2. Subjects and Methods

### 2.1. Participants

This study included 100 Egyptian participants: 30 healthy controls and 70 MS patients with the relapsing–remitting phenotype (RRMS). They were recruited from the Multiple Sclerosis Unit, Ain Shams University Hospitals from May 2020 to February 2021. Thorough medical and neurological assessments were performed by a neurologist according to the 2010 revision of the McDonald criteria [35]. Current or recent inflammatory or infectious disorders in the past month, pregnancy, diabetes, any type of malignancy, or any other neurological condition were all considered excluding factors. RRMS patients were classified into relapse and remission groups; relapse group comprised 35 patients whose samples were taken within 7 days from the onset of relapses and before methylprednisolone therapy, whereas the remission group included 35 patients who had been in a clinical remission state for at least 90 days prior to collection of the samples. Meanwhile, the control group consisted of 30 age-matched apparently healthy volunteers without any history of chronic inflammatory or immunological diseases. The study protocol was approved by the Research and Ethics committee for Experimental and Clinical studies at Faculty of Pharmacy, Cairo University Cairo, Egypt with approval number; BC (2666). Before the study began, all participants were given the necessary information about the study, and their written informed consent was collected. The research was carried out in conformity with the Helsinki Declaration principles, revised in 2008. Disability quantification was calculated using the Kurtzke’s Expanded Disability Status Scale (EDSS) [36]. The annualized relapse rate (ARR), the number of confirmed relapses experienced by the patient in one year, was determined for each patient during the previous two years [37]. Based on the calculated ARR, both relapse and remission groups were subdivided into two subgroups each: either an ARR < 1 or ARR ≥ 1.

### 2.2. Sample Collection and Biochemical Measurements

Five milliliters of venous blood were collected from each participant using BD Vacutainer^®^ SST II Gel collection tubes. The separated sera were aliquoted and frozen at −80 °C for the analysis of lncRNAs and gene expression levels [22,38,39]. An aliquot of the serum was used to assess TGF-β1 and IL-1β levels.

### 2.3. Total RNA Extraction and Reverse Transcription

The miRNeasy Mini Kit (Qiagen, Hilden, Germany) was used to extract total RNA from 200 µL of serum using QIAZOL lysis reagent as per the manufacturer’s protocol. The isolated RNA was dissolved in 50 µL of RNase-free water and kept at −80 °C until it was analyzed. RNA concentration and quality were evaluated using a UV–visible spectrophotometer nanodrop (Thermo Scientific, Waltham, MA, USA). The RNA yield range was 700–1400 nanograms, and the purity range at wavelength 260/280 was 1.8–2.0 [40]. Reverse transcription of RNA into complementary DNA (cDNA) was performed using the RT2 first strand Kit (Qiagen, Hilden, Germany). The RT reaction was incubated for 60 min at 37 °C, followed by 5 min at 95 °C. The produced cDNA was kept at −20 °C until analysis was conducted [22,38].

### 2.4. Quantitative Real-Time Polymerase Chain Reaction (qRT-PCR)

Relative expression levels of lnc-EGFR, SNHG1, and lincRNA-Cox2 along with gene expression levels of FOXP3, NLRP3, caspase-1, and ASC were measured using the RT2 SYBR Green Master Mix kit (Qiagen, Hilden, Germany) as per the manufacturer’s instructions. Glyceraldehyde 3-phosphate dehydrogenase (GAPDH) was used as a housekeeping reference gene. In brief, for the assessment of lncRNAs (lnc-EGFR, SNHG1, and lincRNA-Cox2), 2 μL of cDNA was used as a template in 25 μL of total reaction volume containing 12.5 μL of RT2 SYBR Green PCR master mix, 9.5 μL of nuclease-free water, and 1 μL of RT2 lncRNA PCR primer assay. The primers were created using NCBI primer Blast, verified by the in silico PCR tool of the University of California, Santa Cruz (UCSC) genome browser, and eventually custom made by Invitrogen by Thermo Fisher Scientific. The primer sequences are presented in Supplementary Table S1. qRT-PCR was performed with a Qiagen Rotor Gene Q6 Plex Real-Time PCR system (Qiagen, Hilden, Germany), with a PCR initial activation at 95 °C for 10 min, followed by 40 cycles at 95 °C for 15 s and 60 °C for 60 s. For the evaluation of FOXP3, NLRP3, caspase-1, and ASC relative gene expression, qRT-PCR was performed in a 25 μL reaction mixture prepared by mixing 12.5 μL of master mix, 2.5 μL of primer assay, 5μL of cDNA, and 5 μL of RNAase-free water. The reaction was performed with a PCR initial activation at 95 °C for 15 min followed by 40 cycles at 95 °C for 15 s, 55 °C for 30 s, and 72 °C for 30 s. Data were analyzed with Rotor Gene Q software (Qiagen, Germany) with the automatic threshold cycle (Ct) setting. Then the relative expression for each measured biomarker after normalization to GAPDH as an endogenous control was calculated by applying the 2^−ΔΔct^ method and presented as fold change [22,38].

### 2.5. TGF-β1 and IL-1β Levels Using Enzyme-Linked Immunosorbent Assay (ELISA)

Serum TGF-β1 was assayed using a Proteintech sandwich ELISA kit (Proteintech Group Inc., Rosemont, IL, USA) (Catalog Number: KE00002) according to the manufacturer’s instructions. Meanwhile, serum IL-1β was assessed using a PicoKine™ ELISA Kit (MyBioSource Inc., San Diego, CA, USA) (Catalog Number: MBS175901) as per the manufacturer’s instructions.

## 3. Statistical Analyses

All studied parameters were tested for normality of distribution using a Kolmogorov–Smirnov test. The results were expressed as median and range or mean and standard error of the mean (SEM) whenever appropriate. Normally distributed datasets were analyzed for significance using unpaired Student’s two-tailed t-tests and analysis of variance (ANOVA) followed by post hoc Tukey multiple comparison test. Simple linear regression analysis was conducted using a Pearson χ^2^ test to study the correlation between serum levels of lnc-EGFR, SNHG1, and lincRNA-Cox2 with each other and with the clinical characteristics and biochemical markers in RRMS patients.

For receiver operating characteristic (ROC) analysis, the RRMS group was compared to the healthy control group to evaluate the diagnostic precision of the measured parameters, whereas the relapse group was compared to the remission group to assess the prognostic power of our biomarkers. The area under the curve (AUC) and optimal cut-off values were calculated. The positivity rates were compared using a chi-square test. The overall accuracy of a molecular marker to predict different groups was defined as the average of the sensitivity and the specificity. Univariant binary logistic regression analysis was conducted to predict the potential use of the measured parameters as diagnostic markers for RRMS and prognostic tools for active relapse in RRMS. *p*-values < 0.05 were considered statistically significant. Odds ratios and 95% confidence intervals (CI) were calculated. All data were statistically analyzed using Windows-based SPSS statistical software (SPSS version 20.0, SPSS Inc., Chicago, IL, USA) and GraphPad Prism 9.2 (GraphPad Software 9.2.0, San Diego, CA, USA).

## 4. Results

### 4.1. Demographic and Clinical Characteristics of the Study Participants

The demographic and clinical characteristics of the study participants are summarized in Table 1. RRMS patients in relapse and remission groups exhibited disease duration ranges 2–10 and 1–10 years, respectively, with a median disease duration of 5 years in both groups. The median of the annualized relapse rate in the last 2 years was 1.5 for RRMS subgroups. The median EDSS score for the relapse group was 3, while the median score for the remission group was 2.

### 4.2. Levels of lnc-EGFR, FOXP3, and TGF-β1

**Figure 1:**
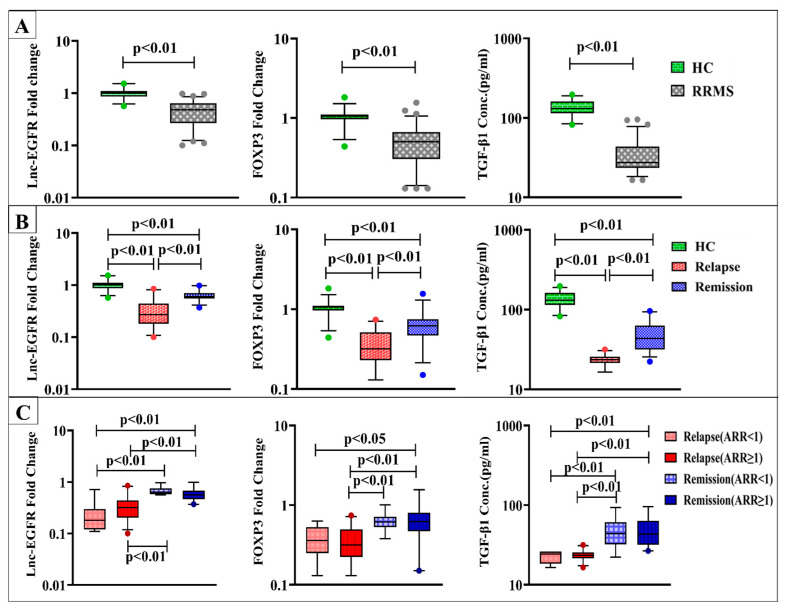
Expression levels of lnc-EGFR and FOXP3 and serum TGF-β1 levels in (**A**) healthy controls (HC) (n = 30) and RRMS patients (n = 70), (**B**) HC (n = 30), RRMS patients during relapse (n = 35), and RRMS patients in remission (n = 35), and (**C**) RRMS patients during relapse and in remission with ARR < 1 (n = 7), (n = 16) and ARR ≥ 1 (n = 28), (n = 19) respectively. Box plots show the median as a band inside each box, while boxes and whiskers delineate 25–75th and 10–90th percentiles, respectively. Dots outside the whiskers indicate outliers. Significant *p*-values are indicated on graph at *p* < 0.01.

RRMS patients showed significantly lower serum levels of lnc-EGFR, FOXP3, and TGF-β1 in comparison to healthy controls at *p* < 0.01 (Figure 1A).

Regarding disease activity, significant downregulation of lnc-EGFR and FOXP3 gene expression along with lower serum TGF-β1 levels were found in RRMS patients during active relapses and in remission compared to healthy control subjects. Notably, patients in the relapse group displayed more significantly reduced levels than those in the remission group at *p* < 0.01(Figure 1B).

When using ARR for subclassification of relapse and remission groups, serum lnc-EGFR, FOXP3, and TGF-β1 levels were not significantly different within the subgroups of both relapse and remission with ARR < 1 or ≥ 1. Meanwhile, both lnc-EGFR and TGF-β1 levels in patients during relapses with either ARR < 1 or ≥ 1 were found to be lower than those in remission with ARR < 1 or ≥ 1 at *p* < 0.01. Additionally, RRMS patients during relapses with ARR < 1 had significantly lower expression levels of FOXP3 than patients in remission with ARR ≥ 1 at *p* < 0.05. While the expression levels of FOXP3 in relapse patients with ARR ≥ 1 displayed significant downregulation than those in remission with ARR < 1 and ≥ 1 at *p* < 0.01 (Figure 1C).

### 4.3. Levels of SNHG1, lincRNA-Cox2, NLRP3, ASC, Caspase-1, and IL-1β

**Figure 2:**
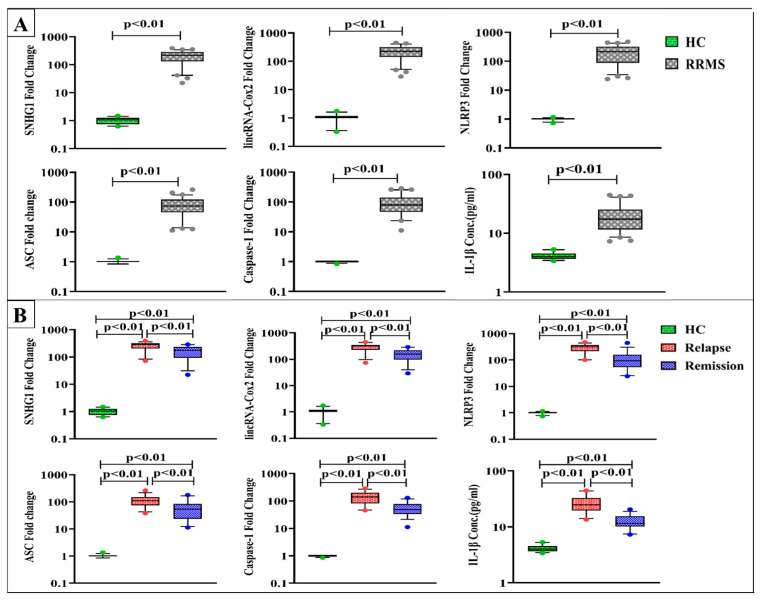
Expression levels of SNHG1, linRNA-Cox2, NLRP3, ASC, and caspase-1 and serum IL-1β levels in (**A**) healthy controls (HC) (n = 30) and RRMS patients (n = 70), (**B**) HC (n = 30), RRMS patients during relapse (n = 35), and RRMS patients in remission (n = 35). Box plots show the median as a band inside each box, while boxes and whiskers delineate 25–75th and 10–90th percentiles, respectively. Dots outside the whiskers indicate outliers. Significant *p*-values are indicated on the graph at *p* < 0.01.

Results in Figure 2A revealed dramatic upregulation in the expression levels of SNHG1, lincRNA-Cox2, NLRP3, ASC, and caspase-1 along with considerable elevation in IL-1β levels in RRMS patients versus healthy controls at *p* < 0.01.

In terms of disease activity, SNHG1, lincRNA-Cox2, NLRP3, ASC, caspase-1, and IL-1β levels were substantially elevated in both relapse and remission groups compared to control values. RRMS patients during active relapses exhibited remarkably higher levels than patients in remission at *p* < 0.01 (Figure 2B).

**Figure 3:**
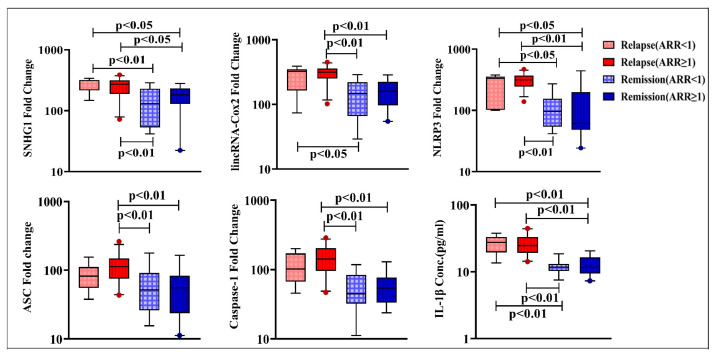
Expression levels of SNHG1, lincRNA-Cox2, NLRP3, ASC, and caspase-1 and serum IL-1β levels in RRMS patients during relapse and in remission with ARR < 1 (n = 7 and 16, respectively) and ARR ≥ 1 (n = 28 and 19, respectively. Box plots show the median as a band inside each box, while boxes and whiskers delineate 25–75th and 10–90th percentiles, respectively. Dots outside the whiskers indicate outliers. Significant *p*-values are indicated on the graph at *p* < 0.05.

Concerning ARR stratifications, SNHG1, lincRNA-Cox2, NLRP3, ASC, and caspase-1 expression as well as IL-1β levels were not significantly different within relapse and remission subgroups with ARR < 1 and ≥ 1. Meanwhile, patients during relapses with ARR < 1 had significantly higher levels of SNHG1, NLRP3, and IL-1β compared to those in the two remission subgroups, though lincRNA-Cox2 levels in this relapse subgroup were found to be higher than the levels of patients in remission with ARR < 1 only. Interestingly, RRMS patients during relapses with ARR ≥ 1 showed significantly elevated levels of SNHG1, lincRNA-Cox2, NLRP3, ASC, caspase-1, and IL-1β versus patients in remission with ARR < 1 and ≥ 1 ((Figure 3).

### 4.4. Correlation Analyses of Lnc-EGFR, SNHG1, and LincRNA-Cox2 Levels

Pearson’s correlation analyses revealed that the expression of lnc-EGFR was negatively correlated with age, number of relapses in the last 2 years, ARR, EDSS score, SNHG1, lincRNA-Cox2, NLRP3, ASC, caspase-1, and IL-1β in RRMS patients. Additionally, lnc-EGFR was positively correlated with FOXP3 and TGF-β1. On the other hand, the expression of SNHG1 was found to be positively correlated with age, ARR, lincRNA-Cox2, NLRP3, ASC, caspase-1, and IL-1β. LincRNA-Cox2 was positively correlated with number of relapses in last 2 years, ARR, EDSS score, NLRP3, ASC, caspase-1, and IL-1β. Moreover, both SNHG1 and lincRNA-Cox2 serum levels were negatively correlated with lnc-EGFR, FOXP3, and TGF-β1, as presented in Table 2.

### 4.5. Diagnostic Potential of the Studied Parameters

**Figure 4:**
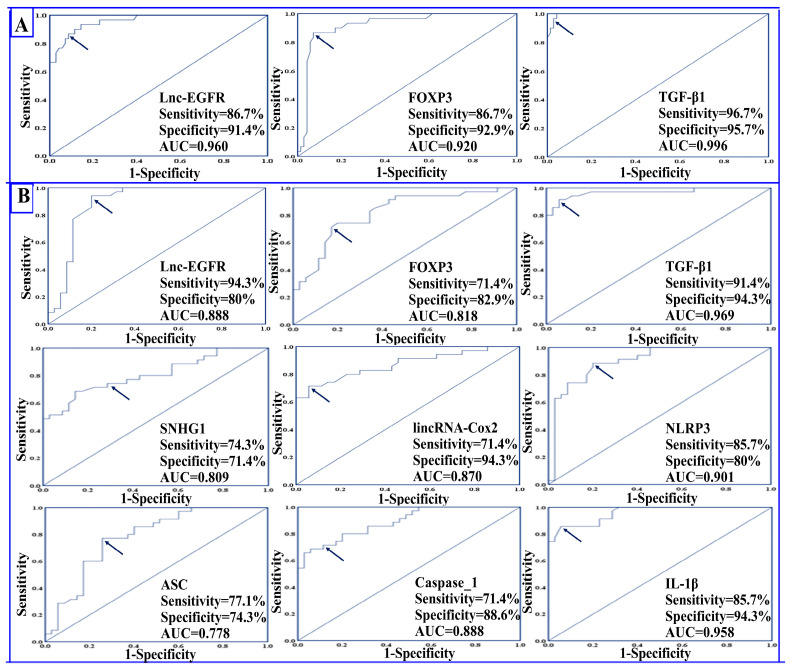
Receiver operating characteristic (ROC) curves showing (**A**) the diagnostic power of lnc-EGFR, FOXP3, and TGF-β1 in differentiating RRMS patients (n = 70) from healthy controls (n = 30) and (**B**) the prognostic power of all the studied biomarkers in differentiating RRMS patients during relapse (n = 35) from patients in remission (n = 35). The arrow denotes the best cutoff point.

ROC curve analysis demonstrated an excellent performance for lnc-EGFR, FOXP3, and TGF-β1 in the diagnosis of RRMS, where the AUCs were 0.960, 0.920, and 0.996, respectively. The positivity rate for lnc-EGFR at the cutoff value 0.78 was 86.7% in the serum of healthy controls compared to 8.6% in the serum of RRMS patients, while the positivity rate for FOXP3 at the cutoff value of 0.83 was 86.7% in serum of controls compared to 7.1% in the serum of RRMS patients. Meanwhile, the positivity rate for TGF-β1 at the cutoff value of 82.45 was 96.7% in the serum of healthy controls compared to 4.3% in the serum of RRMS patients. Interestingly, both lnc-EGFR and FOXP3 had the same sensitivity of 86.7% and had specificities of 91.4% and 92.9%, respectively. The sensitivity and specificity for TGF-β1 was 96.7% and 95.7%, respectively, as shown in Table 3 and Figure 4A.

### 4.6. Prognostic Potential of the Studied Parameters

Data presented in Table 3 and Figure 4B indicate that all the studied parameters exhibited strong potential for differentiating RRMS patients during relapses from those in remission. The AUCs for lnc-EGFR, FOXP3, SNHG1, lincRNA-Cox2, and NLRP3 were 0.888, 0.818, 0.809, 0.870, and 0.901, respectively. The highest prognostic performance was displayed by TGF-β1, where the AUC was 0.969, followed by IL-1β, where the AUC was 0.958. Regarding the positivity rate, lnc-EGFR and FOXP3 had positivity rates of 94.3% and 71.4% in RRMS patients in remission compared to 20% and 17.1% in patients in the relapse state at cutoff values of 0.44 and 0.53, respectively. On the other hand, the positivity rate for SNHG1, lincRNA-Cox2, and NLRP3 were 74.3%, 71.4%, and 85.7%, respectively, in RRMS patients during relapse compared to 28.6%, 5.7%, and 20%, respectively, in RRMS patients in remission at cutoff values of 214.5, 261.3, and 207.3, respectively.

Lnc-EGFR displayed the highest sensitivity of 94.3% followed by TGF-β1 and NLRP3 with sensitivities of 91.4% and 85.7%, respectively. Regarding specificity, lincRNA-Cox2, TGF-β1, and IL-1β all had the highest specificity of 94.3%.

### 4.7. Univariant Binary Logistic Regression Analysis

Univariate logistic regression analysis was employed for the prediction of RRMS (Table 4). Decreased expression levels of lnc-EGFR, FOXP3, and TGF-β1 were designated as significant predictors for the diagnosis of RRMS patients.

In order to predict the progression to the active relapse state, univariate logistic regression analysis was performed between relapse and remission RRMS patients (Table 4). Data indicated that SNHG1, lincRNA-Cox2, lnc-EGFR, NLRP3, ASC, caspase-1, FOXP3, TGF-β1, IL-1β, EDSS score, no. of relapses, and ARR could be used as significant predictors for the progression from a remission state to active relapse.

## 5. Discussion

Relapsing–remitting MS (RRMS) is the most common MS course, which manifests as discrete attacks of neurological dysfunction followed by complete, partial, or no remission. It is believed that clinical relapses reflect acute inflammation in the CNS triggered by flair-ups of autoimmune processes. With time, most relapses become associated with incomplete remission, resulting in permanent neurological disability [5,41]. Hence, further identification of key molecular players underlying relapse incidence might provide novel clues that aid optimal relapse management and delay progression.

To the best of our knowledge, the present study revealed for the first time differential expression of three novel lncRNAs (lnc-EGFR, SNHG1, and lincRNA-Cox2) along with altered expression of some related biomolecules in Egyptian patients with RRMS during active relapses and in remission.

Recently, mounting evidence has indicated that lncRNAs are potential immune system regulators and supported the idea that lncRNAs are implicated in the pathogenesis of autoimmune disorders [24,42]. In the current investigation, the expression levels of lnc-EGFR were found to be significantly downregulated in the sera of all RRMS patients compared to healthy controls. Moreover, patients in the relapse group displayed a more pronounced reduction than those in the remission group. These findings suggest a role for lnc-EGFR in RRMS pathogenesis, especially during disease activity. As far as we know, no previous studies have assessed the expression of lnc-EGFR in MS and no data are available to agree with or contradict our results.

Lnc-EGFR is a newly discovered lncRNA that has been shown to substantially influence the function of Treg cells in HCC [27]. Interestingly, increased expression of lnc-EGFR was observed in HCC that was accompanied by an increased ratio of Treg cells within the tumor micro-environment. Additionally, positive correlation was shown between lnc-EGFR expression and the expression of FOXP3 and EGFR. Moreover, lnc-EGFR, which binds to EGFR specifically, blocks EGFR ubiquitination by inhibiting the interaction between EGFR and ubiquitin ligase, casitas B-lineage lymphoma (c-CBL). As EGFR ubiquitination by c-CBL leads to EGFR degradation, blocking EGFR ubiquitination results in maintenance of EGFR activation, leading to the stimulation of Treg differentiation and thus promoting HCC immune evasion [27]. Of note, FOXP3-expressing Tregs are normally known to actively maintain self-tolerance and immune homoeostasis via their suppressive functions against various immune responses; however, their functions in cancer were found to be co-opted by tumor cells to escape immune surveillance [43].

In MS patients, earlier studies have demonstrated a reduction of functionally effective Treg cells, which was associated with downregulated FOXP3 expression and decreased frequency of FOXP3+ cells [11,44]. Meanwhile, other investigations reported that the reduced suppressive capacity of Treg cells in MS was related to qualitative abnormalities rather than decreased frequency [44].

Herein, the downregulation of lnc-EGFR was accompanied by decreased expression of FOXP3 and reduced levels of TGF-β1 in RRMS patients compared to healthy controls. Additionally, the serum levels of FOXP3 and TGF-β1 were significantly reduced in patients during relapses compared to those in remission state.

FOXP3 is the key transcription factor controlling Treg cell development, differentiation, and immunosuppressive function [7,8]. In agreement with our results, decreased FOXP3 expression in peripheral blood of RRMS patients compared to controls was previously reported [45]. Furthermore, FOXP3 expression was found to be significantly lower in RRMS patients during relapse than in remission [46,47]. Similarly, Ghadiri et al. [48] showed a significant decline in the expression level of FOXP3 in relapsing compared to control groups.

The pleiotropic cytokine TGF-β is a known inducer for the differentiation of Treg cells via induction of FOXP3 expression. Additionally, the secretion of TGF-β by Treg cells, as a soluble mediator of immune suppression, represents one of the suppressive mechanisms to prevent immune activation [49]. However, conflicting data were reported about TGF-β levels in the blood of MS patients. Consistent with the present findings, significantly reduced serum levels of biologically active TGF-β1 were observed during exacerbation of MS [50], and lower levels of TGF-β1 were shown in RRMS compared to healthy subjects [51]. Additionally, a recent study revealed diminished serum levels of TGF-β along with reduced PBMC mRNA expression of TGF-β and FOXP3 in RRMS patients compared to healthy controls [52]. Meanwhile, other studies revealed insignificant changes in TGF-β1 serum levels between patients with RRMS and normal controls [53,54]. On the contrary, significantly elevated serum levels of TGF-β1 were found in RRMS patients that were further augmented during relapse [55]. Similarly, the concentration of biologically active TGF-β1 in plasma from MS patients in relapse were higher compared with those in remission [56]. These discrepancies might be attributed to the small sample size, differences in disease duration/EDDS scores or environmental and ethnic factors in each study.

Former studies indicated that in RRMS patients, relapse frequency, expressed by annualized relapse rate (ARR), correlated positively with long-term physical disability and increased risk of disease progression [57]. Notably, based on ARR subclassification, we observed that patients with both low (ARR < 1) and high (ARR ≥ 1) relapse frequency exhibited significantly decreased lnc-EGFR and TGF-β1 levels during relapses compared with the corresponding subgroups in remission. Meanwhile, FOXP3 expression in patients during relapse with ARR ≥ 1 was significantly lower than patients in remission with both low and high relapse frequency. These outcomes suggest links between the reduced levels of these biomarkers and exacerbation of RRMS and future progression. Indeed, Pearson’s correlation analyses revealed negative correlations between lnc-EGFR expression and number of relapses in the last 2 years, ARR, and EDSS score in RRMS patients.

Additionally, the expression of lnc-EGFR in RRMS patients was positively correlated with FOXP3 and TGF-β1. Such findings comport with the well-recognized roles of these three biomarkers as crucial immune regulators and pointed to a potential role for lnc-EGFR in RRMS. Thus, we could hypothesize that the reduced expression of lnc-EGFR observed here might result in decreased Tregs cell numbers and/or dysregulated Treg cells with impaired immunosuppressive function, as evidenced by the observed low levels of FOXP3 and TGF-β1, which subsequently could participate in the pathogenesis of RRMS, particularly in the immune-mediated events during relapses. Nevertheless, this is the first work to address the involvement of lnc-EGFR in MS, and additional mechanistic studies are needed to confirm this hypothesis.

Upon conducting ROC analysis, lnc-EGFR, FOXP3, and TGF-β1 all displayed excellent diagnostic power for RRMS and strong prognostic potential in differentiating RRMS patients during relapse from those in remission. Moreover, logistic regression analysis showed that decreased levels of lnc-EGFR, FOXP3, and TGF-β1 were designated as significant predictors not only for the diagnosis of RRMS but also for the progression from a remission state to an active relapse. These data may support the involvement of lnc-EGFR, FOXP3, and TGF-β1 in both RRMS pathogenesis and disease activity.

Emerging data emphasizes the involvement of lncRNAs, including the recently discovered SNHG1 and lincRNA-Cox2, in regulating neuroinflammation [58]. The current study is the first to demonstrate concurrent upregulation of SNHG1 and lincRNA-Cox2 in RRMS patients compared to healthy controls with a remarkable increase in their serum levels in RRMS patients during active relapses compared to remission. Our results suggest that both lncRNAs are involved in the pathogenesis of RRMS and reveal a possible relationship between SNHG1 and lincRNA-Cox2 with MS activity.

The observed upregulation of SNHG1 justifies the recent bioinformatics analysis conducted by Sabaie et al. using two microarray datasets of peripheral blood T cells from patients with RRMS and matched controls. These authors identified higher levels of SNHG1 in RRMS patients compared with the controls [31]. Our findings are also in agreement with reports concerning upregulation of SNHG1 in other neurological disorders such as in vitro cell models of Parkinson’s disease (PD) [29] and Alzheimer’s disease (AD) [59], a mouse model of PD [29], and human postmortem brain tissue samples derived from PD patients [28].

It is noteworthy that knockdown of SNHG1 exerted neuronal protective effects and attenuated Aβ25-35-induced neuronal injury in an in vitro AD cell model [59]. Additionally, SNHG1 upregulation promoted microglial activation and enhanced the levels of NLRP3 inflammasome components in activated BV2 microglial cells. Moreover, downregulation of SNHG1 suppressed microglial activation markers and reduced NLRP3, ASC, and cleaved caspase-1 levels in the midbrain tissues of an MPTP-induced PD mouse model [29].

On the other hand, lincRNA-Cox2 has been recognized as a critical component of the inflammatory response. It is highly induced by TLRs and can mediate both the activation and repression of immune genes [60]. Recently, Xue and his colleague have identified lincRNA-Cox2 as a regulator of NLRP3-inflammasome-mediated neuroinflammation. They demonstrated that lincRNA-Cox2 could bind directly to NF-κB p65 to promote its translocation to the nucleus, thus enhancing the expression of the NLRP3 inflammasome sensor and the ASC adaptor. Furthermore, knockdown of lincRNA-Cox2 inhibited the activation of the NLRP3 inflammasome and prevented caspase-1 activation, resulting in decreased IL-1β secretion in activated microglia and macrophages in vitro. Additionally, knockdown of lincRNA-Cox2 improved the clinical outcome of EAE in the mouse model of MS and alleviated neuroinflammation in vivo [34].

Inflammasomes are multiprotein complexes of the innate immune response involved in the activation of caspase-1, the maturation and secretion of pro-inflammatory cytokines IL-1β and IL-18, and the induction of pyroptosis that releases additional inflammatory mediators. Of these, the NLRP3 inflammasome has been described as a critical and necessary mediator in the progression of EAE and MS [12,17]. Apart from its role in inflammation, it is believed that NLRP3 inflammasome activation is associated with disease pathogenesis via other mechanisms such as promotion of pathogenic Th1 and Th17 cell differentiation, recruiting T cells to the CNS, and neurodegeneration [14,15,16,17].

The results of the present study revealed a significant increase in the gene expression levels of NLRP3, ASC, and caspase-1 along with elevated serum levels of IL-1β in RRMS patients compared to healthy controls. Additionally, RRMS patients during active relapses showed significantly higher levels than those in remission. Previous work by Peelen et al. showed upregulated gene expression of the NLRP3 inflammasome components including NLRP3, caspase-1, and IL-1β in PBMCs from RRMS patients in comparison to the control group [18]. Similarly, serum levels of caspase-1 and ASC were higher in MS patients than in the control group [20]. Indeed, the expression of NLRP3, ASC, caspase-1, and IL-1β were reported to be significantly increased in active demyelinating lesion of MS, while their expression levels were greatly reduced in chronic inactive lesions of MS [61]. Furthermore, the gene expression level of the NLRP3 in PBMCs of RRMS patients in remission was found to be significantly lower than that of patients at relapse [62].

In fact, there are no data available about the relationship of the NLRP3 inflammasome with either SNHG1 or lincRNA-Cox2 in MS patients. Herein, we found that the expression of both SNHG1 and lincRNA-Cox2 were positively correlated with NLRP3, ASC, caspase-1, and IL-1β. Additionally, both lncRNAs were positively correlated with each other. These findings comply with the neuroinflammatory roles of SNHG1 and lincRNA-Cox2 in other neurodegenerative diseases and imply that the impact of both lncRNAs on RRMS pathogenesis and disease activity might be mediated, at least partially, via targeting NLRP3 inflammasome activation. Future studies are needed to determine the exact mechanism underlying such activation.

We observed that both SNHG1 and lincRNA-Cox2 serum levels were negatively correlated with lnc-EGFR, FOXP3, and TGF-β1. Such negative correlation may be justified by their opposing role in MS development and might also be related to the recent findings of Park et al., who demonstrated a novel effect for the pro-inflammatory sensor NLRP3 as a negative regulator for Treg cell differentiation through reducing FOXP3 expression [63].

Interestingly, upon stratifying both relapse and remission patients according to their ARR, significantly elevated levels of SNHG1, lincRNA-Cox2, NLRP3, ASC, and caspase-1 as well as IL-1β were observed in patients during relapses with high relapse frequency (ARR ≥ 1) than those in remission with both low (ARR < 1) and high (ARR ≥ 1) relapse frequency. Moreover, correlation analyses showed that both SNHG1 and lncRNA-Cox2 were positively correlated with ARR. Hence, our results suggest an association of these lncRNAs and the NLRP3 inflammasome component levels with exacerbation of RRMS and disease progression.

In this study, ROC analysis showed that SNHG1, lincRNA-Cox2, NLRP3, ASC, and caspase-1 as well as IL-1β exhibit strong prognostic performance in discriminating RRMS patients during active relapses from those in remission. The positivity rates for SNHG1, lincRNA-Cox2, NLRP3, ASC, caspase-1, and IL-1β were 74.3%, 71.4%, 85.7%, 77.1%, 71.4%, and 85.7%, respectively, in RRMS patients during relapse, while they were 28.6%, 5.7%, 20%, 25.7%, 11.4%, and 5.7%, respectively, in patients in a remission state. Additionally, univariate logistic regression analysis revealed that both lncRNAs and all NLRP3 inflammasome elements could be used as significant predictors for the progression from a remission state to an active relapse. These outcomes provide additional confirmation of the usage of SNHG1, lincRNA-Cox2, the NLRP3 sensor, the ASC adaptor, caspase-1, and IL-1β as robust biomarkers for predicting RRMS activity.

In summary, this study demonstrated for the first time altered expression profiles of lnc-EGFR, SNHG1, and lincRNA-Cox2 in Egyptian patients with RRMS. The observed downregulation of lnc-EGFR was associated with decreased expression of FOXP3 and reduced levels of TGF-β1, while the upregulation of SNHG1 and lincRNA-Cox2 were positively correlated with overexpression of NLRP3 inflammasome components. Importantly, more pronounced alterations were found in patients during relapses versus those in remission. The three lncRNAs were correlated with the relapse rate in RRMS patients. These findings suggest that low levels of lnc-EGFR and high levels of both SNHG1 and lincRNA-Cox2 are linked to the pathogenesis of RRMS, disease activity, and future progression. Our study also provides evidence for the potential use of the three lncRNAs as biomarkers for RRMS. Nevertheless, the current study was limited by being a single-center study with a relatively small sample size. Therefore, further multicentered studies on large RRMS cohorts with long-term follow-up are warranted to compare outcomes and monitor progression.

## Figures and Tables

**Table 1:** Demographic and clinical characteristics of the studied groups.

	Groups	Healthy Controls (n = 30)	RRMS Patients		*p*-Value
Parameters			Relapse (n = 35)	Remission (n = 35)	
Age (Years)					
Range		22–45	20–50	19–48	0.106
Median		30	35	32	
Gender:					
-Male n (%)		10 (33.3%)	9 (25.7%)	11 (31.4%)	0.779
-Female n (%)		20 (66.7%)	26 (74.3%)	24 (68.6%)	
MS Family History					
-Negative		\\	33 (94.3%)	33 (94.3%)	0.693
-Positive		\\	2 (5.7%)	2 (5.7%)	
Disease Duration (years)					
-Range		\\	2–10	1–10	0.282
-Median		\\	5	5	
Relapses in Last 2 Years					
Range		\\	2–6	1–5	0.658
Median		\\	3	3	
ARR in Last 2 Years					
-Range		\\	1–3	0.5–2.5	0.672
-Median		\\	1.5	1.5	
EDSS Score					
-Range		\\	1.5–6	1–6	0.04
-Median		\\	3	2	
Treatment: n (%)					
-Interferon-β		\\	25 (71.4%)	23 (65.7%)	0.071
-Fingolimod		\\	10 (28.6%)	12 (34.3%)	

RRMS: relapsing–remitting multiple sclerosis. EDSS: expanded disability status scale. ARR: annualized relapse rate.

**Table 2:** Pearson’s correlation analysis of lncRNA (lnc-EGFR, SNHG1, and lincRNA-Cox2) levels with clinical characteristics and biochemical markers in RRMS patients.

	Lnc-EGFR	SNHG1	lincRNA-Cox2
Age	−0.198 *	0.262 **	N.S.
Disease Duration	N.S.	N.S.	N.S.
Relapses in Last 2 Years	−0.331 **	N.S.	0.298 *
ARR	−0.321 **	0.245 *	0.283 *
EDSS Score	−0.347 **	N.S.	0.322 **
Lnc-EGFR	-----	−0.705 **	−0.648 **
FOXP3	0.721 **	−0.563 **	−0.606 **
TGF-β1	0.733 **	−0.777 **	−0.742 **
SNHG1	−0.705 **	-----	0.706 **
lincRNA-Cox2	−0.648 **	0.706 **	-----
NLRP3	−0.631 **	0.708 **	0.824 **
ASC	−0.584 **	0.649 **	0.594 **
Caspase-1	−0.581 **	0.689 **	0.776 **
IL-1β	−0.717 **	0.692 **	0.768 **

Significant correlation at * *p* < 0.05, ** *p* < 0.01. N.S.: non-significant.

**Table 3:** Positivity rates of the studied biomarkers and overall sensitivity, specificity, PPV, NPV, and accuracy.

I-Between RRMS Patients and Healthy Controls	Healthy Controls (n = 30)	RRMS Patients (n = 70)	Chi-Square X2	*p*-Value	Sensitivity (%)	Specificity (%)	PPV (%)	NPV (%)	Accuracy (%)
**Lnc-EGFR**									
**-No. of +ve cases (≥0.78)**	26 (86.7%)	6 (8.6%)							
**-No. of −ve cases (<0.78)**	4 (13.3%)	64 (91.4%)	58.859	0.000	86.7	91.4	81.25	94.11	89.05
**FOXP3**									
**-No. of +ve cases (≥0.83)**	26 (86.7%)	5 (7.1%)							
**-No. of −ve cases (<0.83)**	4 (13.3%)	65 (92.9%)	62.087	0.000	86.7	92.9	83.87	94.2	89.8
**TGF-β1**									
**-No. of +ve cases (≥82.45)**	29 (96.7%)	3 (4.3%)							
**-No. of −ve cases (<82.45)**	1 (3.3%)	67 (95.7%)	82.362	0.000	96.7	95.7	90.6	98.5	96.2
**II-Between RRMS patients during relapse and in remission**	**Relapse** **(n = 35)**	**Remission** **(n = 35)**	**Chi-Square** **X^2^**	***p*-Value**	**Sensitivity (%)**	**Specificity** **(%)**	**PPV (%)**	**NPV (%)**	**Accuracy** **(%)**
**Lnc-EGFR**									
**-No. of +ve cases (≥0.44)**	7 (20%)	33 (94.3%)							
**-No. of −ve cases (<0.44)**	28 (80%)	2 (5.7%)	39.433	0.000	94.3	80	82.5	93.33	87.15
**FOXP3**									
**-No. of +ve cases (≥0.53)**	6 (17.1%)	25 (71.4%)							
**-No. of −ve cases (<0.53)**	29 (82.9%)	10 (28.6%)	20.902	0.000	71.4	82.9	80.6	74.35	77.15
**TGF-β1**									
**-No. of +ve cases (≥29)**	2 (5.7%)	32 (91.4%)							
**-No. of −ve cases (<29)**	33 (94.3%)	3 (8.6%)	51.471	0.000	91.4	94.3	94.11	91.66	92.85
**SNHG1**									
**-No. of +ve cases (≥214.5)**	26 (74.3%)	10 (28.6%)							
**-No. of −ve cases (<214.5)**	9 (25.7%)	25 (71.4%)	14.641	0.000	74.3	71.4	72.22	73.53	72.85
**LincRNA-Cox2**									
**-No. of +ve cases (≥261.3)**	25 (71.4)	2 (5.7%)							
**-No. of −ve cases (<261.3)**	10 (28.6%)	33 (94.3%)	31.895	0.000	71.4	94.3	92.59	76.74	82.85
**NLRP3**									
**-No. of +ve cases (≥207.3)**	30 (85.7%)	7 (20%)							
**-No. of −ve cases (<207.3)**	5 (14.3%)	28 (80%)	30.328	0.000	85.7	80	81.08	84.85	82.85
**ASC**									
**-No. of +ve cases (≥73)**	27 (77.1%)	9 (25.7%)							
**-No. of −ve cases (<73)**	8 (22.9%)	26 (74.3%)	18.529	0.000	77.1	74.3	75	76.47	75.7
**Caspase-1**									
**-No. of +ve cases (≥98.4)**	25 (71.4%)	4 (11.4%)							
**-No. of −ve cases (<98.4)**	10 (28.6%)	31 (88.6%)	25.963	0.000	71.4	88.6	86.2	75.6	80
**IL-1β**									
**-No. of +ve cases (≥18.4)**	30 (85.7%)	2 (5.7%)							
**-No. of −ve cases (<18.4)**	5 (14.3%)	33 (94.3%)	45.132	0.000	85.7	94.3	93.75	86.84	90

**Table 4:** Univariant binary logistic regression analysis.

Parameters	B	S.E.	*p*-Value	Odds Ratio	95% CI
Biomarkers for diagnosis of RRMS					
Lnc-EGFR	−11.64	2.46	0.000	0.000	0.000–0.001
FOXP3	−6.96	1.33	0.000	0.001	0.000–0.013
TGF-β1	−0.163	0.064	0.012	0.850	0.749–0.964
Biomarkers that predict prognosis of relapse in RRMS patients					
Lnc-EGFR	−9.491	2.18	0.000	0.000	0.000–0.005
FOXP3	−6.62	1.703	0.000	0.001	0.000–0.038
TGF-β1	−0.529	0.143	0.000	0.589	0.445–0.780
SNHG1	0.013	0.003	0.000	1.014	1.007–1.020
LincRNA-Cox2	0.018	0.004	0.000	1.018	1.010–1.026
NLRP3	0.017	0.004	0.000	1.017	1.010–1.024
ASC	0.021	0.006	0.001	1.022	1.009–1.034
Caspase-1	0.038	0.009	0.000	1.038	1.020–1.057
IL-1β	0.480	0.122	0.000	1.616	1.272–2.053
EDSS	1.742	0.825	0.035	5.712	1.135–28.75
Relapses in Last 2 Years	0.737	0.248	0.003	2.089	1.284–3.409
ARR	1.474	0.497	0.003	4.365	1.65–11.56

## Data Availability

The authors confirm that the data supporting the findings of this study are available within the article. The raw data are available from Heba A. Ewida (hebaal-lah.atef@fue.edu.eg) upon reasonable request.

## Supplementary Materials

The following supporting information can be downloaded at: https://www.mdpi.com/article/10.3390/diagnostics13081448/s1, Table S1: Primer sequences used for real-time PCR.
