# Supplementary material for: Altered Lnc-EGFR, SNHG1, and LincRNA-Cox2 Profiles in Patients with Relapsing-Remitting Multiple Sclerosis: Impact on Disease Activity and Progression
Source: Diagnostics (Basel). 2023 Apr 17;13(8):1448. doi: 10.3390/diagnostics13081448 (PMC10137708; doi:10.3390/diagnostics13081448)
Supplement: Supplementary file 1 [file diagnostics-13-01448-s001.zip › diagnostics-2289600-supplementary.pdf]

Supplementary Table 1: Primer sequences used for real-time PCR.

Gene	Sequence
**Lnc-EGFR**	**Forward** 5´- AACACCCTGGTCTGGAAGTACG-3´<br>**Reverse** 5´- TCGTTGGACAGCCTTCAAGACC-3´
**FOXP3**	**Forward** 5´- GGCACAATGTCTCCTCCAGAGA-3´<br>**Reverse** 5´- CAGATGAAGCCTTGGTCAGTGC-3´
**SNHG1**	**Forward** 5´- AGGCTGAAGTTACAGGTC-3´<br>**Reverse** 5´- TTGGCTCCCAGTGTCTTA-3´
**lincRNA-Cox2**	**Forward** 5´- CGGTGAAACTCTGGCTAGACAG-3´<br>**Reverse** 5´- GCAAACCGTAGATGCTCAGGGA-3´
**NLRP3**	**Forward** 5´- GGACTGAAGCACCTGTTGTGCA-3´<br>**Reverse** 5´- TCCTGAGTCTCCCAAGGCATTC-3´
**ASC**	**Forward** 5´- ATCCAGGCCCCTCCTCAGT-3´<br>**Reverse** 5´- GTTTGTGACCCTCGCGATAAG-3´
**Caspase-1**	**Forward** 5´- GCTGAGGTTGACATCACAGGCA-3´<br>**Reverse** 5´- TGCTGTCAGAGGTCTTGTGCTC-3´
**GAPDH**	**Forward** 5´-GTCTCCTCTGACTTCAACAGCG-3´<br>**Reverse** 5´-ACCACCCTGTTGCTGTAGCCAA-3´
